# Undoing the migrant-citizen binomial: reimagining the boundaries of citizenship through acts of solidarity in a southern European City during the COVID-19 pandemic

**DOI:** 10.3389/fsoc.2026.1670998

**Published:** 2026-03-03

**Authors:** Rosa Gatti

**Affiliations:** Department of Political Science, University of Naples Federico II, Naples, Italy

**Keywords:** civil society, COVID-19 pandemic, immigrants, Italy, migration-citizenship nexus, solidarity citizenship, transversal solidarity, volunteering

## Abstract

The COVID-19 pandemic contributed to making national borders more visible and less permeable, reasserting the centrality of citizenship “as the ultimate marker of belonging and solidarity”, and reaffirming the distinction between citizens and non-citizens. In this context, citizenship and national belonging functioned as rigid categories for determining entitlement to protection and assistance. Many states failed to guarantee social rights to all residents, leading to forms of exclusion, particularly for non-citizens. Simultaneously, the crisis sparked the emergence of new forms of civic engagement and solidarity “from below,” enacted by civil society to fill the void left “from above.” Volunteerism flourished supporting those facing food insecurity, evictions, and economic hardship. In some cases, immigrants themselves became central protagonists of these initiatives. This paper contributes to debates on inclusive citizenship and solidarity by jointly analyzing the concepts of citizenship from below and solidarity from below, applying them to a case study of grassroots practices promoted by migrants and citizens in the city of Naples (Italy) during the pandemic. Drawing on long term ethnographic research, it examines how, through the creation of transversal alliances and mutual aid networks, these actors responded to systemic exclusion and economic marginality. Special attention is given to two intertwined pathways: migrant-led initiatives such as the S.E.E.D.S. project, and actions embedded in the urban commons (beni comuni), self-managed civic spaces that became material and symbolic infrastructures of proximity and care. Both trajectories fostered inclusive solidarity capable of contesting institutional boundaries and reimagining belonging. The paper shows how these practices reframed citizenship, challenging, and renewing how participants defined and enacted their civic roles through social relations. The analysis extends the theory of acts of citizenship by demonstrating how social and political participation during crisis—particularly by migrant actors—produced new grammars of recognition and belonging. These findings encourage a rethinking of solidarity, alliances, membership, borders, and citizenship in more inclusive and dynamic terms.

## 1 Introduction

The Italian public welfare system responded only partially to the social problems and needs that emerged during the massive health crisis and the subsequent economic downturn caused by the COVID-19 pandemic. In this context, civil society played a crucial role through a series of grassroots solidarity initiatives involving a wide range of actors—from established NGOs to radical political groups and *ad hoc* networks of individual citizens. These initiatives contributed to a reconfiguration of the concept of citizenship.

The pandemic functioned as a critical juncture, triggering profound transformations in the meaning and functions of democratic citizenship. This phenomenon became particularly evident during the strict lockdown^1^ phases, when the ‘device of citizenship’ (Moro et al., 2022) underwent significant changes in its foundational dimensions: membership as a legal status, belonging as identity, rights and obligations, and practices of participation. While the long-term structural effects of this shift are still unfolding, the crisis revealed the fragility and contestability of citizenship as a political and social construct.

With reference to citizenship as *legal status*—understood as membership in a community and territory—during the COVID-19 pandemic, boundaries were continuously redefined and multiplied at all levels, from global and European, to national, regional and municipal, highlighting their fluid and contingent nature. Concurrently, national borders have become more prominent and less permeable due to mobility restrictions and border closures, which increased the symbolic and practical value of national citizenship.^2^ However, these mobility restrictions during the pandemic did not affect all individuals uniformly; rather, they were contingent on factors such as country of origin and legal status (citizen, temporary resident, asylum seeker, etc.) (Piccoli et al., 2023). In certain cases, exceptions were granted to specific groups on the basis of their legal or professional status, such as medical staff^3^ (Snowden, 2019; Vanderslott and Marks, 2021). In accordance with the broader trend of contemporary migration policies, these restrictions functioned as mechanisms of migrants’ selection (Beine et al., 2016; De Haas et al., 2019). Conversely, their exceptions have contributed to the establishment of new categories of (desirable and undesirable, deserving and undeserving, helpful and unhelpful, useful and useless) travelers (Rushton, 2011). The distinction between citizens and non-citizens not only influenced the management and control of mobility across borders, but also functioned as the criterion for determining who was entitled to protection and care within national borders during a pandemic. This resulted in the repositioning national citizenship as the pivotal indicator of belonging and solidarity (Triandafyllidou, 2020, p. 261). The state measures reflected an oppositional logic, predicated on “a balance between risk, on the one hand, and belonging and solidarity, on the other” (*Ibid.*), mirroring and reinforcing the nationalization of both solidarity and citizenship. At the same time, exceptional inclusion measures—such as the 2020 Italian regularization program and similar initiatives in Portugal—were introduced, justified by public health imperatives (Bonizzoni and Hajer, 2023).

Conversely, the pandemic and the concomitant state of emergency also challenged civil society, fostering innovative forms of civic engagement and solidarity “from below” at the local level.

The proximity initiatives aimed at addressing social, economic, and relational emergencies, as well as health emergencies, contributed to strengthening social ties through locally grounded practices (Moro et al., 2022). In this manner, civil society endeavored to address state deficiencies, which in many cases has failed to generate inclusive solidarity “from above” that ensures equal social rights for all. In several countries, the socio-economic crisis triggered by the pandemic gave rise to forms of collective support and protest at the local scale. These movements frequently transcended national borders and affiliations, thereby establishing networks of solidarity that operated both across international borders and within national borders.

Migrants were among those most severely affected from these circumstances, often finding themselves involuntarily ‘locked’^4^ within national borders due to mobility restrictions.

Concomitantly, novel forms of resistance, self-organization, solidarity, and mutual aid have emerged in migrants’ support and within migrant communities, pursuing a variety of purposes (Della Puppa and Perocco, 2021): facilitating border crossings (Kynsilehto, 2020; Giliberti and Quereilo Palma, 2020) and providing assistance along migration routes (Milan, 2024); opposing the confinement of asylum seekers and refugees in camps and detention centers (Perolini, 2020); coping with unemployment and impoverishment (Thieme and Tibet, 2020); and securing food or other essential goods, as well as providing informal support to individuals facing eviction or food insecurity (Gatti, 2022a). A review of the Italian case reveals that, to address the social problems and needs arising from the COVID-19 pandemic and the social distancing measures introduced to contain it, grassroots mutual aid initiatives were established (Zamponi, 2024). In some contexts, local authorities also played a proactive role in coordinating heterogeneous actors and facilitating resource circulation (Bifulco et al., 2022; Bonizzoni, 2023). In the absence of institutional support, new forms of self-organization and coalitions among ordinary citizens have emerged, even outside traditional organizations.

A salient example was the spontaneous emergence of mutual aid groups at the level of apartment buildings or neighborhoods, which provided assistance and material support to non-self-sufficient individuals and households in quarantine who have fallen into poverty due to the pandemic. This wave of citizens mobilization also involved foreign residents, who contributed through both native organizations and their own ethnic organizations (Gatti, 2022a). In certain instances, foreign citizens engaged in activities organized by the local communities; in others, they spearheaded self-organized initiatives, acting as interpreters and promoters (Gatti, 2022a).

In this paper, an analysis of “from below” solidarity practices undertaken during the pandemic will be conducted with the aim of rethinking borders, belonging, citizenship, and solidarity in an innovative and more inclusive manner.

From a theoretical standpoint, the paper draws primarily on critical approaches to citizenship (see Isin and Nielsen, 2008; Isin, 2009, 2017; Lister, 2003; Stierl, 2016; Turner, 2016) and solidarity (see Agustín and Jørgensen, 2021; Mohanty, 2003; Schwiertz and Schwenken, 2020; Yuval-Davis, 1994, 1997). Additionally, it engages with literature on civic mobilization in times of crisis (Zamponi, 2024) and studies on internal and welfare bordering (Bendixsen and Näre, 2024; Fauser, 2024; Gargiulo, 2024; Bonizzoni and Dimitriadis, 2024), to frame the role of civil society actors—migrants and non-migrants, citizens and foreigners—in emergency contexts.

Empirically, the study analyzes two bottom-up forms of solidarity promoted locally by immigrants or individuals with a migratory background in alliance with native citizens during the pandemic. These initiatives were identified through ethnographic research conducted in Naples, Italy.^5^

The objective of these initiatives was to address the exclusion of socially marginalized and economically vulnerable individuals—both migrants and non-migrants—from public subsidy systems during the initial lockdown period. The inclusive solidarity was enacted through networks of relationships and transversal alliances (Gatti, 2022a), challenging exclusive solidarity structures, and transcending the boundaries of the national social and political community. Consequently, these practices led to a redefined conception of citizenship and belonging.

The present paper focuses on how bottom-up solidarity practices enacted by migrants and citizens in transversal coalitions during the COVID-19 crisis questioned and transformed citizenship, expanding its meaning beyond legal status and formal rights.

The structure of the paper is as follows. The next section examines the concepts of citizenship and solidarity, with particular attention to their manifestations in migratory contexts. This discussion is linked to the debate on the role of solidarity in shaping collective subjects, highlighting how transversal solidarity practices enact citizenship by enabling subjects in different positions to act as citizens. The third section outlines the research strategy. The fourth section delves into the empirical analysis of solidarity initiatives implemented in Naples during the pandemic, focusing on two solidarity infrastructures where Italians and foreigners collaborated. These acts of solidarity—promoted and enacted by migrants in alliance with Italian citizens—questioned and transformed citizenship, reshaping how participants understood and enacted their roles as citizens through social relations and collective action.

The conclusions underscore the contribution of this analysis to expanding the theorization of acts of citizenship (Isin and Nielsen, 2008), moving beyond the domain of political protest to encompass social solidarity. In this manner, the paper presents an alternative and more inclusive conceptualization of citizenship and solidarity.

## 2 Migration, citizenship, and solidarity

The migration phenomenon holds significant relevance in contemporary society. This importance does not derive merely from its numerical incidence, but from the role it plays in current processes of globalization, in the construction and redefinition of borders and boundaries, and in the rethinking of forms of democratic coexistence.

State borders are traditionally conceived as territorial demarcation lines that separate citizens from foreigners, insiders from outsiders, and members from non-members. However, rather than functioning solely as barriers that exclude, borders can be understood as filters that distinguish between what is considered desirable and undesirable, useful and useless, deserving and undeserving (Anderson, 2013). Once external borders are crossed, internal borders emerge through the multiplication of distinctions that define degrees of belonging and differentiated access to rights (Bonizzoni, 2020; Gatti, 2022b).

These processes of internal bordering have become particularly visible during the COVID-19 pandemic, when welfare and administrative boundaries shaped differentiated access to health and social rights and interacted with solidarity practices that challenged these restrictions (Bendixsen and Näre, 2024; Fauser, 2024; Gargiulo, 2024).

The growing presence of immigrants in urban spaces increasingly challenges the nation-based concept of citizenship. It exposes its internal contradictions, redefines its contours, and highlights the multiplicity of its dimensions, making the cleavage between political and social systems more evident. While rights are theoretically equal for all,^6^ their effective exercise in everyday life is shaped by intersecting categories such as nationality, gender, class, religion, and ethnicity, which generate divergent positions and tensions (Gatti, 2022b; Gatti, 2025).

Citizenship, in its formal dimension, provides individuals with a legal status, deriving from the institutional link with the state and full membership in a national community, creating a dichotomy between citizens and non-citizens. Its substantive dimension, on the other hand, enhances the capacity of individuals to act as citizens in practice, making it possible to explore the dynamics of economic and social inclusion/exclusion and the political and cultural centrality/marginality of persons or groups within a given community. From this perspective, even those excluded from formal citizenship may exercise forms of citizenship from below through political action, social participation, or even access to basic services. These actions are capable of redrawing social relations and challenging from below established boundaries of social inclusion and exclusion (Ambrosini, 2016; Bendixsen and Näre, 2024; Fauser, 2024; Gargiulo, 2024; Yuval-Davis et al., 2018).

During the pandemic, such bottom-up practices intersected with exceptional forms of inclusion legitimized by states, such as the 2020 Italian regularization program and similar measures in Portugal, justified on public health grounds (Bonizzoni and Hajer, 2023).

This antinomy between inclusion and exclusion is inherent in citizenship (Balibar, 2012), which “can be both domination and emancipation separately or simultaneously” and to the construction of which both positions of power and resistance can contribute (Isin, 2009, p. 369). Citizenship as legal status is also central to the governance of exclusive solidarity within the national welfare state (Balibar, 2014). National citizenship, by designating who belongs to the national community, defines who can have access to civil, political and social rights (Marshall, 1950) and enjoy the solidarity of the state. Therefore, exclusion from formal citizenship also regulates the boundaries of solidarity. Citizenship represents an “exclusionary basis for building solidarity” with ambivalent effects: “citizenship is necessarily a contradictory force, because it creates an internal space of social rights and solidarity, and thus an external, exclusionary force of non-membership” (Turner, 2000, p. 135 in Schwiertz and Schwenken, 2020, p. 415).

As Schwiertz and Schwenken point out, solidarity partly mirrors and shares “the ambivalence of citizenship as a concept of both submission and emancipation” (2020, p. 406). Similar to citizenship, which has been called a “slippery concept” (Riley, 1992, p. 180) due to its semantic density, solidarity has been described as a “floating signifier” (Laclau, 1990, p. 20 in Schwiertz and Schwenken, 2020, p. 409; see also Agustín and Jørgensen, 2021) because of its “fundamentally different and potentially conflicting meanings” (Schwiertz and Schwenken, 2020, p. 409), often used in an evocative and normative manner with limited analytical precision (Bauder and Juffs, 2020 in Caciagli, 2021, p. 251).

The commonalities between the two concepts of citizenship and solidarity are not limited to the definitional challenges. Like citizenship, solidarity is also understood as relational, spatial and contentious (Agustín and Jørgensen, 2021).

As a relational practice, solidarity is: (1) generative of political subjectivities and collective identities; (2) inventive of new imaginaries; (3) situated in time and space (Agustín and Jørgensen, 2021).

Solidarity, like citizenship, is characterized by a certain antinomy. It is possible to distinguish between a solidarity “from above” and a solidarity “from below.” The former, institutionalized mainly within the nation-state and supranational entities—such as the European Union^7^—is conceived as an exclusive and exclusionary solidarity, closely linked to the idea of nationhood and reciprocity among national citizens. The latter, by contrast, emerges in the horizontal relations that occur daily among individuals of different origins and with different interests and perspectives. It is conceived as a more inclusive solidarity, capable of overcoming national exclusions and including subjects who do not formally belong to a given nation-state and its imagined community (Anderson, 1991).

In recent decades, migration flows have accelerated and renewed these forms of solidarity from below, both transnational and transversal (Gatti, 2022a).

Recent research on civic mobilization during emergencies (Zamponi, 2024) highlights how mutual aid networks and solidarity coalitions complemented institutional responses during the COVID-19 crisis.

In the wake of the acceleration of asylum-seeker flows that swept across Europe and the United States since 2015, forms of solidarity in favor of migrants—along migration routes and within national borders—have been criminalized by radical right-wing forces (Ambrosini, 2020a,b; Battisti and Bruno, 2023; Giliberti, 2017; Tazzioli and Walters, 2019), transforming practices of humanitarian support practices into ‘political acts of solidarity’ (Rygiel, 2011; Schwiertz and Schwenken, 2020). Forms of solidarity involving migrants have not only developed across state borders but also within them, between differently positioned actors, allowing for transcending and reconfiguring internal borders and belongingness (or rather belonging/togetherness) (Bendixsen and Näre, 2024; Fauser, 2024; Gargiulo, 2024).

The present analysis focuses on this second form of solidarity from below—the transversal one—which connects differently positioned subjects in a situated place and time.

According to Yuval-Davis (1994, 1997), the fundamental principle of transversal solidarity is based on recognizing differences and fostering dialogue among subjects positioned differently within social hierarchies. In light of the social inequalities and power relations in which these differences are reproduced, she suggested the idea of “transversal politics.” Building on this reflection, Lister (2003, p. 81) argues that realizing a “politics of solidarity in difference” is essential to bridge divides and counter the exclusions they generate.

Lister (2007) further defines solidarity as “the capacity to identify with others and to act in unity with them in their claims for justice and recognition” (Kabeer, 2005, p. 7), framing it as a core value of inclusive citizenship and reflecting its horizontal conception. In turn, Mohanty (2003) introduces the concept of “political solidarity” to highlight the challenges inherent in developing solidarity practices within societal power relations and global inequalities. Notwithstanding their differences, the contributions of Mohanty, Yuval-Davis, and Lister converge on a key point: solidarity practices do not cancelling or flattening differences but uses them as a starting point for building unity toward common goals practices of solidarity do not out or differences, but rather employing them as a starting point for the building of unity towards common goals.

Expanding this debate, Schwiertz and Schwenken (2020, p. 414) complement the notion of ‘transversal solidarity’ with that of ‘inclusive solidarity’, which “reveals how new relationships, socialites and collective subjectivities emerge.” These concepts enable us to imagine new forms of commonality in differences, whereby differently positioned individuals join forces to address shared concerns, thereby challenging the prevailing national solidarities that systematically exclude non-citizens through practices confined to the narrow community of national citizens.^8^ To further connect citizenship and solidarity, Schwiertz and Schwenken (2020) propose the concept of ‘solidarity citizenship’, which foregrounds relations between differently positioned subjects, an aspect that often overlooked in citizenship studies. This concept emphasizes how individuals in similar and/or different social situations relate to and care for each other, whether in the context of formalized institutions, such as trade unions, or in the case of transversal practices of inclusive solidarity that emerge from below through informal relationships.

Solidarity citizenship first and foremost highlights *who* participates and is included in solidarity relationships, networks and communities, and *the ways in which*, through solidarity practices, relationships and institutions, social relations and citizenship are challenged and transformed (Schwiertz and Schwenken, 2020).

In the following pages, through two different transversal practices of inclusive solidarity that emerged from below, I will show how the practice of acting in solidarity, implemented in Naples during the COVID-19 pandemic, foster relationships between differently positioned subjects–national citizens and non-national citizens–who, by challenging the *status quo*, acted *de facto* as citizens.

Bottom-up forms of solidarity enact citizenship by enabling non-citizens to act as citizens. Acts of solidarity, understood as forms of activist citizenship, can therefore be conceptualized as ‘acts of citizenship’ (Isin and Nielsen, 2008), broadening the spectrum of this concept beyond political protest, where it has traditionally found greater application.

The following pages will illustrate how these practices and relations of solidarity demonstrate that citizenship can be exercised outside legal frameworks, contributing to its redefinition and extension toward a more inclusive and practice-oriented conception. These theoretical perspectives inform the empirical analysis by framing solidarity practices as acts that challenge exclusionary citizenship and create inclusive forms of belonging. This paper contributes to citizenship studies by extending the concept of acts of citizenship beyond protest, framing solidarity practices as transformative acts that challenge exclusionary logics and generate inclusive belonging.

## 3 The research strategy

The research aimed to explore how bottom-up solidarity practices enacted during the pandemic reconfigure citizenship and belonging. The findings presented in this article are based on a long-term ethnography conducted in Naples. The ethnographic material collected is part of a broader research that aimed to reconstruct the civic and political participation of immigrant population in the local public sphere.^9^ The extensive material collected provides the context in which the proposed reflections were developed. The data was collected in two distinct phases: the first phase, spanning from March 2020 to July 2021, coincided with the peak of the global pandemic, where online observation and interviews were conducted; the subsequent phase, from December 2023 to June 2024, entailed a follow-up investigation into solidarity practices and their evolution. In the initial phase, I engaged in active participation in the online meetings of the Immigrants’ Table of the City of Naples (Tavola degli Immigrati della Città di Napoli), and conducted in-depth interviews with its participants, who were the protagonists of the solidarity initiative under discussion. During the period spanning from December 2023 to June 2024, I expanded the scope of my research on forms of solidarity during the pandemic to encompass additional types of cross-cutting solidarity that had not been previously analyzed.

In-depth interviews were conducted with representatives of two collective actors engaged in direct solidarity actions during the pandemic in Naples, Italy.

From the point of view of their social and political background, the collective actors belong to pre-existing social networks that have adapted their action to the pandemic context, challenging welfare and administrative boundaries that define internal borders (Fauser, 2024; Gargiulo, 2024). The first actor–belonged to the Commos^10^ of Naples has mainly political objectives, sharing a background and values that can be traced back to the radical left. It has long invested in mutual aid as a strategy to rebuild bonds of social solidarity among the neighborhood population. While the second—raised from the associations participating in the Naples Immigrants’ Table (for more details, see Gatti, 2024)—has a composition that is in some ways more heterogeneous than the first, being composed of leaders of ethnic associations and representatives of trade unions and native or mixed organizations dealing with immigration in the Neapolitan area, whose exponents do not share common political values. Therefore, unlike the former, they have no explicit political orientation and aims. Finally, both actors implemented *ad hoc* initiatives in response to the pandemic, focusing on the provision of food-aids to people in need in the city in coordination with municipal actors who facilitated resource circulation among heterogeneous networks (Bifulco et al., 2022; Bonizzoni, 2023).

## 4 Pandemic and solidarity. Building transversal alliances to combat exclusion

The COVID-19 pandemic produced severe social and economic consequences in Italy. In the first three quarters of 2020, employment decreased (−470,000 units; −2.0% compared to the same period of the previous year), with fixed-term jobs (−394.000, −12.9% on average in the first three quarters) and self-employment (−162.000, −3%)^11^ most affected (Istat, 2021). There has been a worsening of the employment status of the most vulnerable categories–women (employment rate of −1.3% compared to −0.7% of men), young people (employment rate of −1.8% for the under 35 s compared to −0.8% of the 35–49 year olds and −0.3% points for the over 50s) and foreigners (for whom the value of the employment rate falls below that of Italians)—experienced the sharpest decline.

During the COVID-19 pandemic in Italy, the number of people in absolute poverty increased (Istat, 2021). Among immigrants, absolute poverty reached one million five hundred thousand foreign residents^12^ (29.3%, compared to 7.5% among Italians) (Istat, 2021).

The generalized *lockdown* introduced on March 10, 2020 (Prime Ministerial Decree of 9 March 2020) and the closure of most commercial activities exacerbated pre-existing inequalities, particularly for those in irregular or precarious work (Ambrosini et al., 2020). Institutions attempted to mitigate these effects through targeted welfare measures, including the ‘Municipal Solidarity Fund’ (Ordinance No. 658/2020), which financed food vouchers for families most exposed to the economic impact of the pandemic. This fund is intended to provide financial support for urgent measures concerning ‘food solidarity’^13^ with the aim of ensuring the availability of essential provisions to Italian municipalities. However, the broad discretion granted to municipalities in defining eligibility criteria often resulted in discriminatory practices,^14^ excluding non-EU citizens, irregular migrants, and individuals without official residence.

By calling into question the issues of the protection of rights and (un)equal access to resources and services, the ‘food solidarity’ measures and their effects highlight how the pandemic crisis has contributed to exacerbated the preexisting inequalities—formal and substantial—already existing in Italian society, including those between citizens and non-citizens, EU citizens and non-EU citizens, and regular and irregular migrants.

These dynamics illustrate how welfare bordering and administrative discretion reinforced internal boundaries of citizenship, determining unequal access to essential resources (Fauser, 2024; Gargiulo, 2024).

In response, civil society actors—namely, activists and community leaders—mobilized to implement more inclusive forms of bottom-up solidarity.^15^ These bottom-up initiatives sought to overcome exclusionary logics and create transversal alliances between migrants and native citizens. Such practices can be interpreted as acts of citizenship, challenging formal boundaries and enacting alternative models of belonging (Isin and Nielsen, 2008). The following section focuses on the case of Naples, analyzing two grassroots solidarity experiences in which migrants played a leading role alongside Italians. These forms of transversal solidarity, operating within national boundaries, emerged as a potential alternative citizenship model.

### 4.1 The participation of migrants in social solidarity infrastructures in the city of Naples (Italy): the commons and the S.E.E.D.S. Project

To deal with the social emergency triggered by the COVID-19 pandemic, the Municipality of Naples established a Municipal Solidarity Fund, which allocated a €300.00 spending bonus to each family unit experiencing financial hardship.^16^ A segment of the Fund, designated ‘Il Cuore di Napoli’ (The heart of Naples), supported by private donations, financed the distribution of 3,123 food parcels, amassed and meticulously catalogued in a designated area within the ‘Mostra d’Oltremare’^17^ trade fair and distributed through the collaborative efforts of Third Sector Associations.^18^ For the allocation of the spending bonus, legal residence was required (municipal resolution no. 91/2020), excluding individuals without official registration—superseding the criteria for *real condition of economic need* and/or *de facto stable residence* in the municipal territory, as stipulated by the Civil Protection (ordinance no. 658/2020). Consequently, many migrants who had lost jobs and lacked family support networks were excluded from receiving bonus. Institutional solidarity has thus revealed its limitations, reinforcing the logic of exclusion and welfare bordering, where administrative criteria create internal boundaries of citizenship and unequal access to resources (Fauser, 2024; Gargiulo, 2024).

Concurrently, solidarity has emerged from below, across the municipality. In response, civil society mobilized through neighborhood committees, social cooperatives, Catholic organizations, community initiatives, and individual volunteers. In the face of socio-economic challenges and experiences of exclusion, these communities have endeavored to adopt more inclusive daily proximity practices, seeking to mitigate the impact of institutional deficiencies. During the period of the Coronavirus emergency, their contribution was particularly relevant,^19^ constituting a genuine manifestation of subsidiary welfare. Migrants and individuals with a migratory background also participated not only as beneficiaries but also as volunteers in these local initiatives, assuming active roles in solidarity infrastructures. The solidarity actions in which they were involved can be categorized essentially into two distinct types: those organized by the native civil society and in which they participated as volunteers, and those organized by the migrants themselves and their ethnic organizations in which they assumed a leading role. Such practices can be interpreted as acts of citizenship, challenging formal boundaries and enacting alternative models of belonging (Isin and Nielsen, 2008).

The ensuing discussion is predicated on the findings of the research conducted. In the following pages, the focus will be on two different bottom-up solidarity actions. The first is an initiative organized by the Commons of Naples, and the second is an initiative organized by the representatives of associations, who permanently participate in the Immigrants’ Table of the Municipality of Naples,^20^ united in the S.E.E.D.S. (Saisir l’égalité puor echapper à la destruction de la societé/Seizing Equality to Escape the Destruction of Society) project.

#### 4.1.1 Common goods and territorial mutualism: Naples faces pandemic

In the summer of 2016, the Municipality of Naples identified several properties under its jurisdiction that had previously been occupied by citizens and associations. These properties were subsequently recognized as spaces “by their own vocation (territorial location, history, physical characteristics) (...) of civic and collective use.” This designation was made in accordance with Council Resolution no. 446/2016, which also acknowledged these properties “for their value as (...) common goods^21^ emerging and perceived by citizens as environments for civic development.” These public properties had been in a state of abandonment and severe degradation for years. Residents have transformed these places from unusable and dilapidated to places “capable of creating social and relational capital in terms of collective uses with the value of common goods” (ibidem). This initiative, initiated in 2012 in the historic city center with the Filangieri nursery school (l’Asilo Filangieri), was subsequently expanded to encompass other areas of the city.

As delineated in council resolution 446 of 2016, ‘Common Goods’ (Beni Comuni)^22^ are defined as spaces that vary significantly in terms of their origins and historical evolutions. However, they are unified by the initiation and expansion of pathways for democratic participation, which have been extended to the entirety of the citizenry, encompassing both Italian citizens and foreign residents. These spaces, overseen by disparate collectives, function as laboratories for grassroots citizenship and the formation of political subjectivity. Beginning in March 2020, with the proliferation of the novel Coronavirus (COVID-19) pandemic and the subsequent declaration of the first lockdown, numerous collectives operating within these spaces underwent a process of self-organization, both as independent entities and through the establishment of networks. These collectives initiated a variety of support and mutual-help initiatives, designed to assist individuals and families grappling with the health emergency and the subsequent social and economic crisis. The experiences observed include the food collections carried out by the collectives of ‘Scugnizzo Liberato’ (Liberated Brat)^23^ with the ‘Scugnizzo solidale’ (‘Solidarity Brat’) initiative, and ‘Sgarrupato’ (Demaged)^24^ with the ‘Spesa sociale (Social Shopping) initiative. In both cases, the initiative started informally with a spontaneous collection of food and crowdfunding to purchase basic necessities. Subsequently, ‘food parcels’ were distributed to families and individuals in need in the Montesanto/Avvocata neighborhoods.

*“*(…) *Since the Coronavirus epidemic broke out, many people have lost their jobs, been fired, closed their businesses or can no longer earn a living as they did before. The measures put in place by the institutions are insufficient: they tell us that it is necessary to stay at home, but many families have serious economic difficulties and the problems that already existed have worsened. The emergency and its political management are highlighting the contradictions of our system. In particular, the distribution of wealth, never as unequal as now: many people cannot economically resist the emergency... We must demand a society in which we live in the name of the common good! While they privatized public healthcare and transformed everyone's health into a business for the few... Meanwhile, mutual aid networks and solidarity initiatives were born in the neighborhoods of Naples to face the emergency together. Like Scugnizzo Liberato, we are taking action to support people in difficulty in the Montesanto and Avvocata neighborhoods with the delivery of shopping parcels of basic necessities. Naples responds to the emergency situation, it does so with the courage that distinguishes it, in an attempt to correct the many distortions of the way of life that we have been collectively forced into. For now, building relationships and support networks is certainly the best way to support each other through hardship. The subsequent phases of the emergency will call us all to imagine a fairer world, in which our rights come first, made for the life of all, not for the interest of the few: the world of life and not that of death. If you need assistance or know someone who might need it, write to us on this Facebook page or call us on XXX (from 9am to 1pm) and XXX (from 4pm to 7pm).*

If you want to support us, leave a donation in the XXX stores, you will allow us to buy pasta, milk, legumes, tomatoes, rice and thus prepare a complete shopping package or make a donation and support us remotely. Everyone must mobilize, each doing their part, identifying responsibilities and spreading solidarity.” (Appeal from Scugnizzo Liberato activists published on Facebook)

The appeal of the Common Goods Network articulates a dual intervention: it denounces the structural inequalities deepened by the COVID-19 pandemic and critiques the institutional responses, perceived as disconnected from the lived experiences of those in conditions of socio-economic precarity. This socio-political critique demands two things: first, grassroots action, and second, collective imagination toward a more just and inclusive society, transcending existing divisions. In this context, the solidarity from below initiatives undertaken by Neapolitan collectives during the initial stages of the pandemic manifest as both relational and political acts, situated and generative. These initiatives, typified by mutual-aid networks centered on the distribution of essential goods, not only addressed immediate material needs to counter the social and economic emergency, but also sought to envision a more equitable world. They did so by proposing an alternative model of citizenship based on the common good and mutual care ethics. Drawing upon principles of inclusiveness and egalitarianism rooted in left-wing political culture, on which their action is based, these networks facilitated spaces of social and political participation for individuals with migratory backgrounds allowing them to feel, live and act as citizens in everyday life.

The glue for being together and common action was the spatial proximity, commonalities related to neighborhood life and universalist values, as well as a collective experience of vulnerability brought about by the health emergency produced by the pandemic. In this sense, solidarity from below functioned as both a response to systemic neglect and a generative force for reimagining citizenship and belonging.

“…*I took part in the occupation of Sgarrupato... Sgarrupato is my thing, it’s practically in my house, downstairs, in front of my apartment... I look out from the balcony, and I see it... I lived there, I still live there, that’s my neighborhood* (emphasis) *... and there we collected and distributed food and ran various fights...* (at the beginning of the pandemic) *I was the only foreigner* (to be part of the organizing committee), *and then I don’t feel like a foreigner… I have never felt different… because they* (collective) *have a different openness towards the Other …there is fertile ground…”* (Ukrainian woman, 42 years old, domestic worker, Sgarrupato volunteer, resident in Italy for over 20 years; interviewed on 17 June 2024)

In this context, the COVID-19 pandemic, as a disruptive external event, has also provided foreign citizens with the opportunity to exercise their capacity for initiative and step out of the ordinary, participating in an extraordinary collective enterprise as citizens-solidarists. The collected narratives not only reflect the immediate impact of the health emergency but also demonstrate the ability of individuals, particularly migrants, to play an active role in establishing networks for providing aid and distributing food. In this framework, the emergence of ‘solidarity citizenship’ subverts the exclusionary dynamics of formal belonging. Identification comes through participation, territorial rootedness, and openness to others, not through legal status.

*“… when the pandemic started, everyone was locked in their homes and I started painting the walls and ceilings of the house, because the time available was excessive and you didn't know where to put your energy... how to release it... but it didn't satisfy me... since I knew a gentleman from a very large association - with which I had already done another project ... - that's how we met - ... when the pandemic began he called me and said "listen, I have 2,000 euros, I would like to invest it to help people (in difficulty)” and naturally I immediately contacted “my people” at Sgarrupato and said to them “guys, there is this possibility, do we want to start? Let's have a meeting, clandestine..." because we were in full lockdown let's have this meeting and initially, they were perplexed because they said "2,000 euros is not much..." I instead told them "let's start! Let's see how it goes, let's exploit it, then let's see who joins..." ... so we made a work plan, which included putting flyers everywhere, on Facebook, but also attached* (on the street), *with my telephone number: people had to call me and give their details, say how the family unit was made up to book the shopping. So my phone number was circulating. I ended up receiving phone calls from more than 800 families, whose details I took. I started making a list of people and families (who were in need). And we started distributing the shopping with that first 2,000 euros.*” (Ukrainian woman, 42 years old, domestic worker, Sgarrupato volunteer, resident in Italy for over 20 years; interviewed on 17 June 2024)

The testimony of a Ukrainian volunteer—a resident for over 20 years and involved in the Sgarrupato mobilization—shows how the pandemic activated the dimension of everyday political action: from spontaneous fundraising to large-scale distribution of basic needs, to building relationships of trust and mutual recognition. Her account exemplifies the transformation of individual initiative into collective project.

*“Then we started to publicize the initiative, asking for an appeal from well-known people/influencers. And the people of the neighborhood began to respond: the fathers of the family, the blacksmith, the butcher, the simple people, everyone brought either some money or some product, some butcher the meat, some greengrocer the fruit, my bakery friend churned out bread to die for. Every grocery bag had fresh bread every time. These 800 families were divided into 6 days. To tell you how much solidarity there was. There were those who helped distribute the shopping on their scooters, those who donated their time... then we put their credit card number on Facebook* (for crowdfunding) *and so people started donating but not 5-10 euros, there was someone from France who donated 3000 euros... it was a scary thing... too beautiful a machine was set in motion*...” (Ukrainian woman, 42 years old, domestic worker, Sgarrupato volunteer, resident in Italy for over 20 years; interviewed on 17 June 2024)

*“A boy made nice big maps with all the houses marked, everyone had the task of bringing the shopping to a family - he knew where he had to go -... then there was the preparation of the parcels. 120-130 bags were made per day. Every day, until they left us a little free* (easing of restrictive measures) *and people started working again and requests started to decrease a little*...” (Ukrainian woman, 42 years old, domestic worker, Sgarrupato volunteer, resident in Italy for over 20 years; interviewed on 17 June 2024)

The excerpt documents how a veritable infrastructure of solidarity was launched with minimal resources and the active participation of more than 800 families. This infrastructure was supported through spontaneous donations, crowdfunding, and local material contributions. Initially informal, the initiative benefited from self-organized logistics, including detailed maps, task division, and the daily production of over one hundred food parcels. At a later stage, in collaboration with the NGO Emergency,^25^ the initiative was further structured and expanded while maintaining operational autonomy through the parallel project, Spesa Sociale.

*“… From March to October 2020 we carried on alone. Then at the end of October Emergency took over and we started collaborating with Emergency, who asked me for the list of people with their telephone numbers and all the information I had, they interviewed the people and decided who to admit (to the aid). However, we at Spesasociale have nevertheless supported for more than a year those who had not entered due to the requirements established by Emergency. In parallel, those who did not have the support of Emergency had that of Spesasociale. They were two different things, but they went in parallel, on different days. They distributed once a week, on Saturdays. While we gave the package during the week to those who hadn't returned. But we didn't have a specific day, they came whenever they wanted to the Sgarrupato, because there was always someone in the Sgarrupato and always the shopping ready on the floor and as soon as someone came, they were given the shopping. And we worked with Emergency for another year and a half”* (Ukrainian woman, 42 years old, domestic helper, Sgarrupato volunteer, resident in Italy for over 20 years; interviewed on 17 June 2024)

In this sense, solidarity from below is not just an emergency response to an institutional vacuum, but a laboratory of new forms of belonging, mutual care and re-imagined citizenship, where even the traditionally excluded find a space to feel, live and act as a citizen in everyday life. In this process, migrant subjects emerge as protagonists, not only as beneficiaries, but also as co-organizers and agents of care. The pandemic opened up an exceptional space in which political action was embodied in everyday practices that generated new forms of belonging. Citizenship, acted and not simply conferred, has been defined through active participation, reciprocity, and self-organization. This counters exclusionary logics and creates fertile ground for a transformative vision of urban living. In this sense, solidarity from below is not merely an emergency response to an institutional void; it is also a laboratory for new forms of belonging, mutual care, and reimagined citizenship. Even the traditionally excluded can find a space to feel, live, and act as a citizen in everyday life.

#### 4.1.2 S.E.E.D.S. as a social infrastructure of solidarity and citizenship from below

The S.E.E.D.S. (Saisir l’égalité puor echapper à la destruction de la societé/Seizing Equality to Escape the Destruction of Society) project is a paradigmatic example of social infrastructure based on self-organized solidarity. This initiative emerged from the mobilization of migrant and interethnic associations in response to the social emergency engendered by the pandemic. The novel aspect of this initiative, which sets it apart at a local level, lies in the active involvement of migrant communities. These communities serve as the source of inspiration and the driving force behind the initiative’s organization. In this case, the foreign communities are not merely recipients of aid but also promoters of mutualistic and collective care practices. They mobilize and act autonomously with respect to local institutions to respond to the needs of foreign families at risk of poverty and social exclusion. These practices have the capacity to identify emerging needs and provide concrete, inclusive responses, even in the absence of institutional support.

The initiative was inspired by the personal commitment of a young Ivorian-Italian woman. She is the president of the interethnic association *Hamef onlus*, which was established to address the needs of immigrant communities. From the onset of the emergency, the president received numerous requests for help from members of the association. She immediately took action to deal with the emergency.

In the initial phase, she initiated the distribution of food informally through her association, leveraging her personal network to secure support and resources. Subsequently, confronted with the urgent appeals for assistance from migrant communities, she sought the support of the Department of Social Policies and Labor of the Municipality of Naples. As a representative of her association and a member of the Immigrants’ Table, she endeavored to establish a collaborative practice of inclusive solidarity with the municipal institution.

*“Knowing the reality of our territory - here people work illegally! – I expected what then happened. So I called the Councilor to tell her what my staff and I were doing, so as to convene the Immigrants' Table and try to work together, because we must not leave anyone behind. The Councilor did not respond to my requests... silence... so I continued with my network, because the families continued to call me”* (Ivorian-Italian women, 40 years old, president of the Hamef Association, resident for over 20 years in Italy; interview on 28 January 2021).

Confronted with the lack of response from the municipal administration, the president of *Hamef Onlus* takes initiative independently. Through the informal WhatsApp group comprising the participants of the Immigrants’ Table of the Municipality of Naples, she identifies and acquires allies to advance the solidarity initiative in support of individuals excluded from institutional aid. The immigrant associations *of* immigrants^26^ were the first to respond to her, subsequently associations *for* immigrants were added.^27^ While the former represented the means to reach foreign communities and families in difficulty, the latter contributed to covering the initial costs.

“I volunteer. How can I help all these people, with children, newborns without diapers? I didn't know how to do it. So, I called Slow Food and other large organizations and every week they started coming with a truck to bring food and I called the representatives of the associations, and they came down to my building and loaded the cars with packages, each group did this... I was in contact with everyone.

After the second week of the total closure... I realized that there were some women with children who couldn't come to the center to get food... Slow food asks me - do they have Postepay? If yes, ask for the card number and we will top up you with 50.00 euros per woman!

*The network is important... my partners made collections within their organizations, I gave the card number, and they recharged it, so the women could go to the supermarket and buy all the stuff for the children. We did so* (Ivorian-Italian women, 40 years old, president of the Hamef Association, resident for over 20 years in Italy; interview on January 28, 2021).

This first informal organization on a voluntary basis was valorized in a short time thanks to the membership, intervention and support of ActionAid,^28^ which allowed the structuring of the S.E.E.D.S. project (Saisir l’égalité puor echapper à la destruction de la societé/Seizing Equality to Escape the Distruction of Society).

“*While I was doing all this personally within my association, ActionAid Napoli contacted me and asked me what I was doing. I was already doing distribution with my network. I told them what I was doing, and they said -Okay! Let’s structure what you are doing! -. They too are within the Immigrants' Table. We all already know each other. We had meetings and wrote the project. We called it* S.E.E.D.S*., “Semi (in Italian)”, which has become a really important project in the area*” (Ivorian-Italian women, 40 years old, president of the Hamef Association, resident for over 20 years in Italy; interview on, January 28, 2021).

The S.E.E.D.S. project it represented an emergency intervention carried out by an extended partnership, made up of immigrant and native associations that join the institutional table of immigrants of the Municipality of Naples and supported thanks to a fundraiser to continue to support families in the most acute phase of the crisis.

The S.E.E.D.S. project constituted a bottom-up proximity circuit that amalgamated disparate realities, unified by a shared objective: the provision of material assistance to Italian and foreign families grappling with adversity and at risk of social exclusion, excluded from the purview of institutional support measures. The organization’s primary objective was the distribution of food at home in the Vasto and Forcella neighborhoods. Additionally, the organization endeavored to provide support to individuals at risk of social exclusion and to small agricultural producers in the Neapolitan area, who were severely impacted by the pandemic due to the closure of local markets. The collaboration with Slow Food^29^ has facilitated the direct procurement of fruit, vegetables, and other essential food products from farms that have been adversely affected by the pandemic. The direct involvement of aid beneficiaries in the preparation and distribution of food parcels, the bottom-up proximity network, has also contributed to achieving the symbolic objective of sowing seeds for a more supportive and inclusive social and political community.

The implementation of the S.E.E.D.S. project underscored the pivotal role of immigrant associations as active contributors to society, adept at intercepting and responding to the exigencies of the territory during times of crisis (Saggiomo, 2020). These associations have also been able to enhance the quality of life for their individual members and foster social cohesion.

The experiences implemented in the response to the epidemic of the novel coronavirus (SARS-CoV-2) have highlighted the crucial role of diaspora associations and their representatives in promptly identifying requests and responding to the needs of the most difficult-to-reach vulnerable communities by guaranteeing targeted interventions on the field. The provision of support for local associations by larger organizations,^30^ as exemplified by ActionAid in this case, occurred subsequent to the initiation of independent action in its nascent stages, thereby enhancing and expanding the scope of the action already undertaken.

“*Thanks to ActionAid we are able to support our people. Every week we bring a shopping bag. Whoever calls us, we try to help them. How many thanks arrive on WhatsApp! People confide in each other. I was told - Tania, I had been at home for three days without even having a crumb of bread to eat, I didn't know what to eat and I was thinking of taking my own life -. A person who stays here, with their country far away, cannot return because the borders are closed, they cannot go out to work, they are locked in their house and are thinking of taking their own life. This period was truly destructive.*

*Instead, we gathered and saw that this distancing brought us even closer together. We understood how important it is to be together. Even if we don't make money. Before maybe someone said: “We stay here without earning a penny, we do a lot of things and then in the end for what? – Instead, now we have all understood that it is important to be together*” (Belarussian-Italian women, 40 years old, president of the Bellarus Association, resident for over 20 years in Italy; interview on 9 July 2020).

As the protagonists’ words reflect, migrants and citizens establish a “social infrastructure of solidarity” (Schilliger, 2020, p. 537) based on a range of social and emotional relationships, forms of sociability, and specific ways of relating to others that help build commonality in difference and provide forms of mutual support. The active involvement of migrant women in distribution and logistics has engendered novel forms of leadership, thereby transforming need into action and action into social ties and a sense of belonging.

As Schilliger (2020, p. 536) observes, examining the effects of a specific solidarity infrastructure “in action” entails not only analyzing the experiences of the involved actors but also examining how it generates new spaces and facilitates novel forms of citizenship “from below.” Project effectiveness is derived from the intertwining of direct beneficiary participation, multi-associative collaboration, and the shared values of social justice, inclusiveness, and caring for others. These forms of “solidarity infrastructure” address urgent material needs and create new spaces of sociality and new grammars of citizenship. In these spaces, belonging is not legitimized by legal status but by collective action. In this sense, S.E.E.D.S. embodies a transformative vision of citizenship, realizing a political laboratory in which migrants become active agents of cohesion and change.

## 5 Rethinking citizenship-migration binomial through the acts of solidarity

The cases analyzed highlight that solidarity is not necessarily based on a pre-established, homogeneous and exclusive community. On the contrary, it can arise through the mediation of differences, with respect to legal status, class, race and gender, and through the formation of new transversal subjectivities, capable of including differently positioned subjects in collective daily practices. These social formations and the acts they stage allow us to re-discuss the terms of mutual recognition, belonging and citizenship.

However, it is important to acknowledge that these networks are not exempt from internal tensions or limitations. Scarce resources, unequal access, and power asymmetries can generate conflicts and challenge their ability to sustain inclusive practices over time (Schwiertz and Schwenken, 2020).

The forms of bottom-up solidarity implemented during the pandemic jointly by migrants and citizens are configured as forms of bottom-up citizenship, and specifically as a type of solidarity citizenship (Gatti, 2022a), as they not only facilitated the recognition and inclusion of non-citizens, but they also allowed us to “weave relationships and build institutions of solidarity potentially capable of contributing to social cohesion and a redefinition of citizenship on a larger scale” (Schwiertz and Schwenken, 2020, p. 406).

The subjects involved in the solidarity initiatives analyzed become political subjects in collective action, transforming themselves from simple ‘active citizens’, as they are already civically engaged in their organizations, into ‘activist citizens’ (Isin, 2009, 2012), who create new forms of solidarity and demand rights, recognition and belonging.

If acts of citizenship are defined, among other things, as those acts in which “ways of becoming political are actualized or executed” (Isin and Nielsen, 2008, p. 39), the cases analyzed show that, not only acts of an intrinsically political nature, but also acts of a predominantly social nature, such as acts of affiliation and solidarity, can be configured as acts of citizenship.

The stimulus to collective action in solidarity, to “hold together with each other,” came from the observation of the common condition of vulnerability that emerged with the pandemic and at the same time from the social and political exclusion based on the origin suffered in its emergency phase. If on the one hand, the pandemic has brought about a transformation of (identity)citizenship, shifting the attention from belonging to a “community of origin,” in which common socio-cultural traits are shared on a national basis, to belonging to a “community of destiny,” as can also be seen from the messages circulated “together we will make it,” “we are all in the same boat”; on the other hand, however, this “common destiny” has not prevented the perpetuation of exclusion and social injustice from a formal point of view on the basis of possession of the legal status of citizen. Precisely this antinomy—between the feeling of belonging and the common destiny, on the one hand, and the exclusion and injustice suffered, on the other hand—represented the stimulus to action, to act in solidarity with a purpose that was at the same time redistributive and reclaiming.

Being with others with a common objective of solidarity starting from different positions represented for the subjects involved not only participation in a social formation but also belonging to a transversal political alliance, and the instrument through which to claim recognition. This last aspect is even more evident in the case of subjects with a migratory background involved in the S.E.E.D.S. project. In fact, the women–migrants and non-migrants–who joined the S.E.E.D.S. project used the results obtained–*we are supporting more than 250 families, we do not want pity*–during an institutional meeting of the Immigrants’ Table (Gatti, 2024), to claim their right to count and legitimize their counter-discourse focused on agency, capability and citizenship in opposition to those who describe them as victims and passive, in which vulnerability and compassion are used as a norm to regulate the relationship with the other.

Through a performative exercise, they produce themselves as subjects capable of acting as solidarity citizens, creating a transversal alliance capable of providing an alternative solution to a collective problem. In this way they implement not only a social but also a political action, which is both redistribution and recognition (Fraser, 2000; Gatti, 2024).

At the same time, the initiatives developed within Naples’ Commons—such as Scugnizzo Liberato and Sgarrupato—played a similarly transformative role. These spaces, recognized under municipal resolution no. 446/2016 as civic-use properties, functioned as platforms of bottom-up citizenship well before the health emergency. During the pandemic, they acted as territorial infrastructures of solidarity, enabling spontaneous mobilization, resource sharing, and the construction of inclusive mutual-aid networks. Their strength resided not only in material assistance, but in their capacity to activate political subjectivation across citizenship status, fostering transversal alliances that redefined the terms of belonging.

In these urban commons, proximity, relational density and shared values served as catalysts for collective action, uniting native residents and migrants around practices of care and rights-claiming. The notion of solidarity from below—within both the Commons and the S.E.E.D.S. project—emerges as a powerful tool for rethinking citizenship as an active, situated and inclusive process.

Solidarity produced from below, through political and affective alliances, outside the claims of similarity and commonality (see Butler and Athanasiou, 2019, p. 167), offers a space within which to dismantle social conventions and structures of exclusion, make visible and audible (Gatti, 2024) and overcome the boundaries of national belonging.

Looking at these practices–both oppositional (Hooks, 2020) and generative–as the result of a constellation of social and political factors allows us to recognize migrant and citizen actors as critical political subjects capable of challenging established boundaries and social labels.

If acts of citizenship are evaluated on the basis of their transformative outcomes, then only one of the two cases analyzed fully exhibited the characteristics of acts of citizenship (see Isin and Nielsen, 2008). However, jointly analyzing citizenship and solidarity in migratory contexts through the lens of transversal alliances expands the scope of citizenship acts theory beyond mobilizations of undocumented populations, encompassing broader civic, social, and institutional participation.

In the aftermath of the acute phase of the pandemic, the solidarity practices described have shown divergent trajectories. However, in both cases there has been a loss of momentum, attributable to scarce resources, internal conflicts and the individual paths of volunteers. However, after the emergency phase, during which these practices were supported by structured third sector organizations such as Action Aid and Emergency, none of them were institutionalized through partnerships with local authorities. Moreover, local welfare services did not incorporate them into their practice, using them as exemplars of best practice. In the case of Common Goods, a form of grassroots solidarity persists, albeit in a different form. In contrast, the S.E.E.D.S. project has reached its conclusion. This situation highlights the challenge of maintaining solidarity beyond emergency contexts and requires further investigation into the conditions that enable the continuity and strengthening of solidarity infrastructures.

It is crucial to emphasize that Naples is not an isolated case in which civil society has assumed a leading role in managing the emergency posed by the novel coronavirus. It is evident that there are other documented cases that demonstrated parallels with Naples, most notably that of Milan (see Bonizzoni, 2023; Bifulco et al., 2022), where the proactive role of local authorities in facilitating the circulation of resources through the involvement of a broad and diverse coalition of civil society actors has been highlighted. As was the case in Naples, this coalition comprised actors from the radical left and professionalized third sector organizations. However, in contrast to the Neapolitan case, where these networks operated independently and in response to the absence of municipal action (Gatti, 2024), in the Milan case, the networking of these actors and the circulation of resources among them was facilitated by the municipality in collaboration with non-governmental organizations such as Emergency. Despite the differences between the two cases, it appears that public authorities can indirectly rely on the action of civil society, which operates in a subsidiary capacity, and utilize it to reach individuals who would otherwise be excluded from aid. This strategy gives rise to questions regarding the management, design and redesign of internal boundaries, and the individuals with the capacity to challenge and re-signify them.

The acts of solidarity enacted by citizens and non-citizens in Naples during the pandemic have the potential to reverse the conventional migrant-citizen binary and reimagine citizenship boundaries within national borders in a more inclusive manner.

Concurrently, the divergent trajectories that the practices analyzed highlights the challenge of maintaining solidarity beyond emergency contexts and give rise to questions regarding the temporality of acts of citizenship (Isin and Nielsen, 2008) and their capacity to engender enduring institutional change.

Future research endeavors could profitably expand upon this analysis through the implementation of comparative studies in various urban or national contexts (Ataç et al., 2021). These comparative studies could explore the role of local institutions, the composition of solidarity networks, and their capacity for institutionalization. The aim of these studies would be to verify whether analogous dynamics of citizenship and solidarity emerge in diverse sociopolitical configurations. The objective of these comparative studies is twofold: first, to ascertain any discrepancies and commonalities among social response models, and second, to provide a comprehensive analysis of the aforementioned models and their applications.

## Data Availability

The datasets presented in this article are not readily available because the full interview transcripts contain sensitive data, and, in agreement with the interviewees, in signing the authorization for use, there is no provision for transfer to third parties. Requests to access the datasets should be directed to rosa.gatti@unina.it.

## Footnotes

1 In this context, the term “lockdown” refers to the measures of confinement—that is, the reduction of mobility and interpersonal contacts—that are adopted in order to contain the spread of the virus, in accordance with the protocol that was adopted by the World Health Organization in 2020. China was the first to implement such measures, following the examples of South Korea and Italy, and subsequently other countries, by restricting the movement of its citizens within national borders and the entry of foreigners from other countries. Consequently, states were compelled to adopt maximum security measures.

2 Indeed, in the context of the COVID-19 pandemic, national citizenship allowed people to return to their countries. However, the picture is fluid: some countries—including Australia and Morocco—have prevented their citizens from entering, thus potentially acting in violation of their international human rights obligations (see Hicks, 2021; Piccoli et al., 2020a, 2020b, 2023; Piccoli et al., 2021). Moreover, other statuses have emerged as crucial, such as residency. In this regard, the role played by legal residency for both nationals and immigrants legally residing on Italian territory is relevant, both for intra-national mobility, in the case of commuters (both students and workers), and for access to food subsidies, in the case of vulnerable groups (both Italian and foreign), as described in the following paragraphs.

3 With reference to the Italian case, in March 2020 at the height of the *lockdown*, Chinese, Russian and Cuban doctors arrived in Italy to support the collapsing Italian healthcare system.

4 The word “locked” refers to the condition of immobility experienced by numerous migrants during the pandemic. These migrants found themselves trapped in the settlement country, unable to return to their countries of origin or move freely within the host country. They were confined to their homes due to mobility restrictions. Most of these individuals are employed in manual occupations and are unable to work remotely, which has resulted in job loss and the experience of severe economic vulnerability.

5 The results of the research conducted in 2020–2021 were integrated with those collected during a follow-up conducted in 2023–2024, as described in Section 3.

6 Citizenship is regulated according to a stratified and multi-scalar legal system, which arranges foreigners in a kind of pyramid (cf. Ambrosini, 2016, p. 86), according to which different categories of immigrants correspond to different legal conditions, resulting in differentiated access to different types of rights.

7 Solidarity represents one of the founding values of the European Union, defined in the EU Treaty of Lisbon.

8 It should be pointed out that in many European societies fundamental human rights apply to everyone, including noncitizens, and that some welfare systems guarantee forms of social solidarity even to noncitizen foreigners. However, it is also true that there is a discrepancy between the formal level of norms and the substantive level of their application, and exclusions related to special conditions occur, such as what happened in the case described.

9 The focus of the research was the process of mobilization and visibility of female component of migrant population. Using multiple techniques and analytical tools, I identified and followed 22 migrant women from different geographical backgrounds who, by playing the role of leaders within migrant organizations, “won the competition for access to the public sphere and (...) made themselves known locally” (Mantovan, 2007, p. 117). I followed these women in their spaces of participation both offline and online (Caliandro, 2018), since their everyday practices of citizenship took place not only outside but also within the digital space, which became indispensable with the onset of the Covid-19 infection and related legislative measures, which restricted freedom of movement, encounter and participation. For the results of the entire research, see Gatti (2025).

10 In the Neapolitan context, Commons are urban spaces abandoned and subsequently reclaimed by citizens through practices of self-management and civic regeneration. Since 2011, groups of citizens have identified disused assets-such as municipal buildings, industrial areas and confiscated land-organizing themselves into horizontal communities to return them to public use. This process had the character of civil disobedience but led to a collective and inclusive valorization of the territory. Naples institutionalized this vision, creating a Department for Common Goods and including the legal concept of “common good” in the municipal statute (for more detail see: https://commonsnapoli.org/, Accessed January 24, 2025). In the Neapolitan case, we are dealing with “urban commons of tangible and accidental former public goods (abandoned municipal buildings, urban sites, industrial areas, neighborhood cultivable areas or confiscated assets) that can activate social micro-arenas and forms of government mutualistic and cooperative” (see Vittoria and Mazzarella, 2021).

11 The worst trends are to be found in the hotel and restaurant sector and in domestic services (female-dominated), among trade and service workers and among unskilled occupations.

12 The pandemic has had a negative effect especially on the economic conditions of foreign households: in fact, 26.7% of households composed exclusively of foreigners and 25.3% of households with at least one foreigner live in absolute poverty (compared to 6.0% of households with only Italians) (Istituto Nazionale di Statistica, 2021).

13 The initial allocation for the 2020 Municipal Solidarity Fund was 400 million euros, subsequently increased with the Prime Ministerial Decree of 28 March 2020.

14 There were many Italian municipalities in which potentially discriminatory criteria have been adopted. In many cases, which were then legally contested it involved discrimination against foreign citizens who were not legally resident. The Courts of Rome, Ferrara, Brescia and Naples consulted entered into the merits of the conditions set by the Municipalities for the provision of ‘food vouchers’, establishing the illegitimacy of the requirement of the EU permit for long-term residents and of residence in the Municipality or in any case on the national territory (which also presupposes the possession of a residence permit) and condemning the Municipalities to repeat the procedure by modifying the criteria and to compensate the excluded for the damage by granting them the denied bonus.

15 A survey carried out in Italy (Corvo et al., 2022) on a total of 563 Italian third sector entities (ETSs) revealed that only one-third of the organizations were requested to collaborate with the public sector to mitigate the negative effects of the pandemic. In other cases, spontaneous support initiatives were undertaken by third sector entities to manage the crisis in a supplementary role relative to the public administration.

16 According to the Report on accountability of the Munipality of Naples Rapporto sulla rendicontazione del Comune di Napoli (2020), about 10.5% of the resident population (total residents: 1,003,466 people; about 100 thousand beneficiaries) took advantage of the bonus activated by the Municipality. Since the data disaggregated by nationality are not reported, it is not possible to determine how many foreigners have (or not) benefited from municipal subsidies.

17 The Mostra d’Oltremare is the largest exhibition space in Naples, extending over a total of 50 thousand square meters between outdoor and indoor spaces.

18 It should be noted that dialogue, connection and coordination with the municipal administration has been difficult to identify forms of connection and coordination, also with regard to the issuance of free movement authorizations for carrying out volunteer activities during the lockdown, creating several problems for the organization of aid by volunteers.

19 The Commons alone assisted 1,700 families and raised over 80 thousand euros.

20 The Table of Immigrants of the Municipality of Naples includes not only immigrant associations, but also mixed-composition associations and associations composed mainly of natives working in the field of immigration.

21 Commons are defined as “tangible and intangible assets of collective belonging which are removed from the logic of exclusive use and characterized by shared and participatory management. The Municipality of Naples guarantees the collective use of common goods and their preservation for the benefit of future generations, through a public government that allows their fair and supportive use. The Municipality of Naples is the first city in Italy to have established a Department of Common Goods to give strength to the theme of the forms of use of heritage for the prevailing collective interest”, we read on the Municipality’s website (https://www.Comune.napoli.it/flex/cm/pages/ServeBLOB.php/L/IT/IDPagina/16783) to which please refer for further information on the action of the Municipality of Naples in favor of the institution of the Commons.

22 Commons are seven: Villa Medusa, the former Lido Pola in Bagnoli, the former Opg (former S. Eframo Nuovo Monastery) and the Giardino Liberato (former Teresian Convent) in Materdei, the former Conservatory of Santa Fede (Liberata) and the Scugnizzo Liberato (former Filangieri prison, former Convento delle Cappuccinelle) in the historic center together with the former Schipa in via Salvator Rosa.

23 The word ‘scugnizzo’ is a Neapolitan term for a lively, cunning and intelligent ‘street brat’. And it represents a symbolic figure of the insurrection against the German troops during the 4 Days of Naples of 27–30 September 1943. Many were in fact the ‘scugnizzi’ who took part in this well-known episode of Italian resistance for liberation from Nazi occupation. ‘Scugnizzo Liberato’ (Liberated Brat) is a ‘Common Good & Mutual Aid Laboratory’ in the city of Naples located at Salita Pontecorvo 46, in the city centre.

24 The term ‘sgarrupato’ is a Neapolitan term for something that is damaged, ruined, decayed, dilapidated. ‘Sgarrupato’ is a (previously ruined) building in the densely populated Montesanto district, which was salvaged more than 25 years ago by activists and locals, subsequently used for social activities for children and young people and during the lockdown period for the distribution of food and medicine to the poor in the neighbourhood. ‘Sgarrupato’ is a mutual aid organization active in the same area of Naples in which the ‘Scugnizzo Liberato’ operates, with which it collaborates on various activities.

25 Emergency is a non-profit NGO founded in 1994 to provide free, high quality medical and surgical treatment to victims of war, landmines and poverty. It promotes a culture of peace, solidarity and respect for human rights. It is recognized as an NGO partner of the United Nations—Department of Public Information and part of the Economic and Social Council as an association in Special Consultative Status.

26 The Senegalese Association of Naples, the Bellarus Association, the Nigerian Vivlaviv Association and the Italian-Gambian Association.

27 The Uniti Campania Association (Italian Union for Immigrants), which represents the structure of the Uil Campania union which deals with issues relating to immigration.

28 ActionAid is an independent international organization, founded in 1972 and present in 71 countries around the world, acting against poverty and injustice together with the most discriminated against communities.

29 Slow Food is an international non-profit movement founded in 1986 by Carlo Petrini in Italy with the aim to promote local food traditions, protect biodiversity, and encourage sustainable food practices. The movement serves as a counter to fast food culture, advocating for the enjoyment of food that is good, clean, and fair. Slow Food emphasizes the importance of respecting the environment and local ecosystems while valuing the knowledge and traditions of food producers (www.slowfood.it).

30 With the aim of improving the interventions carried out by migrant associations in response to the pandemic, IOM Italia has financed six projects thanks to the support of the Ministry of Foreign Affairs and International Cooperation (MEACI-DGCS) and the Italian Agency for Cooperation to Development (AICS).
